# Physiology of the Respiratory Drive in ICU Patients: Implications for Diagnosis and Treatment

**DOI:** 10.1186/s13054-020-2776-z

**Published:** 2020-03-24

**Authors:** Annemijn H. Jonkman, Heder J. de Vries, Leo M. A. Heunks

**Affiliations:** 1 Department of Intensive Care Medicine, Amsterdam UMC, Location VUmc, Amsterdam, The Netherlands; 2 Amsterdam Cardiovascular Sciences Research Institute, Amsterdam UMC, Amsterdam, The Netherlands

## Abstract

This article is one of ten reviews selected from the Annual Update in Intensive Care and Emergency Medicine 2020. Other selected articles can be found online at https://www.biomedcentral.com/collections/annualupdate2020. Further information about the Annual Update in Intensive Care and Emergency Medicine is available from http://www.springer.com/series/8901.

## Introduction

The primary goal of the respiratory system is gas exchange, especially the uptake of oxygen and elimination of carbon dioxide. The latter plays an important role in maintaining acid-base homeostasis. This requires tight control of ventilation by the respiratory centers in the brain stem. The respiratory drive is the intensity of the output of the respiratory centers, and determines the mechanical output of the respiratory muscles (also known as breathing effort) [1, 2].

Detrimental respiratory drive is an important contributor to inadequate mechanical output of the respiratory muscles, and may therefore contribute to the onset, duration, and recovery from acute respiratory failure. Studies in mechanically ventilated patients have demonstrated detrimental effects of both high and low breathing effort, including patient self-inflicted lung injury (P-SILI), critical illness-associated diaphragm weakness, hemodynamic compromise, and poor patient-ventilator interaction [3, 4]. Strategies that prevent the detrimental effects of both high and low respiratory drive might therefore improve patient outcome [5].

Such strategies require a thorough understanding of the physiology of respiratory drive. The aim of this chapter is to discuss the (patho)physiology of respiratory drive, as relevant to critically ill ventilated patients. We discuss the clinical consequences of high and low respiratory drive and evaluate techniques that can be used to assess respiratory drive at the bedside. Finally, we propose optimal ranges for respiratory drive and breathing effort, and discuss interventions that can be used to modulate a patient’s respiratory drive.

## Definition of Respiratory Drive

The term “respiratory drive” is frequently used, but is rarely precisely defined. It is important to stress that the activity of the respiratory centers cannot be measured directly, and therefore the physiological consequences are used to quantify respiratory drive. Most authors define respiratory drive as the intensity of the output of the respiratory centers [3], using the amplitude of a physiological signal as a measure for intensity. Alternatively, we consider the respiratory centers to act as oscillatory neuronal networks that generate rhythmic, wave-like signals. The intensity of such a signal depends on several components, including the amplitude and frequency of the signal. Accordingly, we propose a more precise but clinically useful definition of respiratory drive: the time integral of the neuronal network output of the respiratory centers, derived from estimates of breathing effort. As such, a high respiratory drive may mean that the output of the respiratory centers has a higher amplitude, a higher frequency, or both.

The respiratory drive directly determines breathing effort when neuromuscular transmission and respiratory muscle function are intact. We define breathing effort as the mechanical output of the respiratory muscles, including both the magnitude and the frequency of respiratory muscle contraction [1].

## What Determines the Respiratory Drive?

### Neuroanatomy and Physiology of the Respiratory Control Centers

**Fig. 1:**
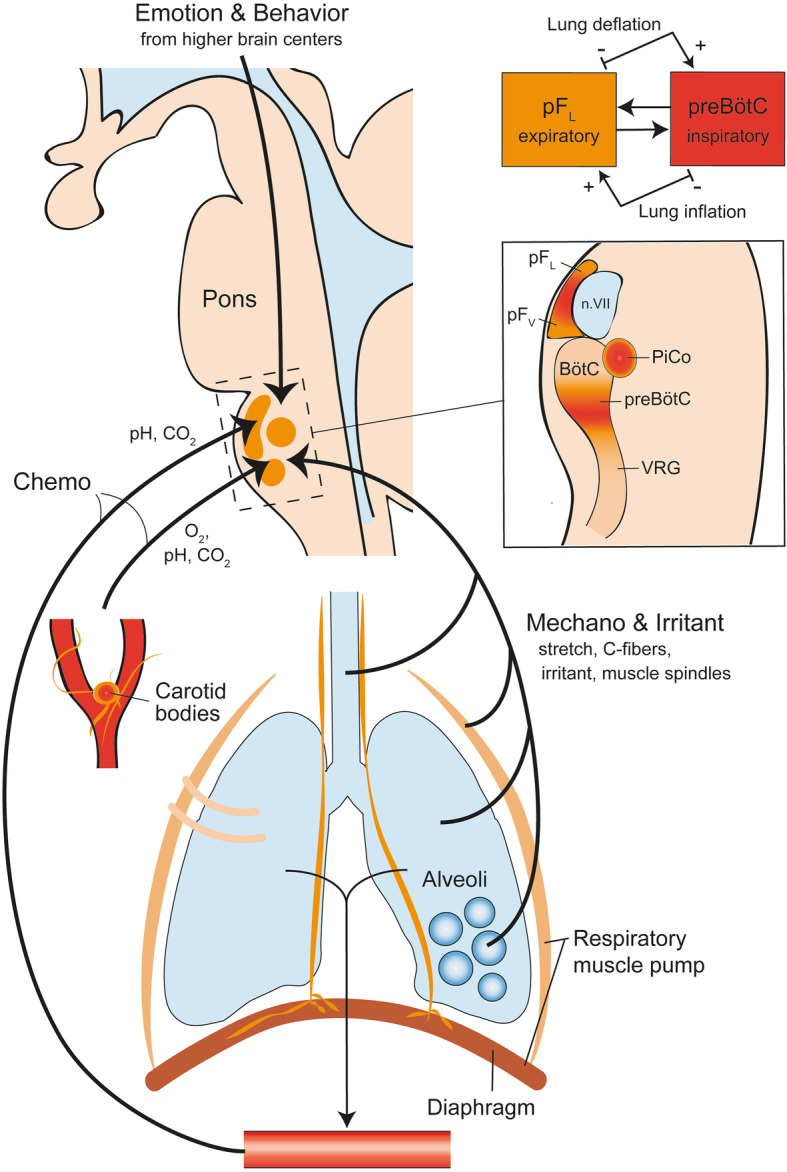
Schematic representation of the anatomy and physiology of respiratory drive. The respiratory centers are located in the medulla and the pons and consist of groups of interneurons that receive information from sources sensitive to chemical, mechanical, behavioral, and emotional stimuli. Important central chemoreceptors are located near the ventral parafacial nucleus (pF_V_) and are sensitive to direct changes in pH of the cerebrospinal fluid. Peripheral chemoreceptors in the carotid bodies are the primary site sensitive to changes in PaO_2_, and moderately sensitive to changes in pH and PaCO_2_. Mechano and irritant receptors are located in the chest wall, airway, lungs, and respiratory muscles. Emotional and behavioral feedback originate in the cerebral cortex and hypothalamus. The pre-Bötzinger complex (preBötC) is the main control center of inspiration, located between the ventral respiratory group (VRG) and the Bötzinger complex (BötC). The post-inspiratory complex (PiCo) is located near the Bötzinger complex. The lateral parafacial nucleus (pF_L_) controls expiratory activity and has continuous interaction with the pre-Bötzinger complex, to prevent inefficient concomitant activation of inspiratory and expiratory muscle groups: lung inflation depresses inspiratory activity and enhances expiratory activity, which ultimately results in lung deflation. Lung deflation has the opposite effect on these centers

**Fig. 2:**
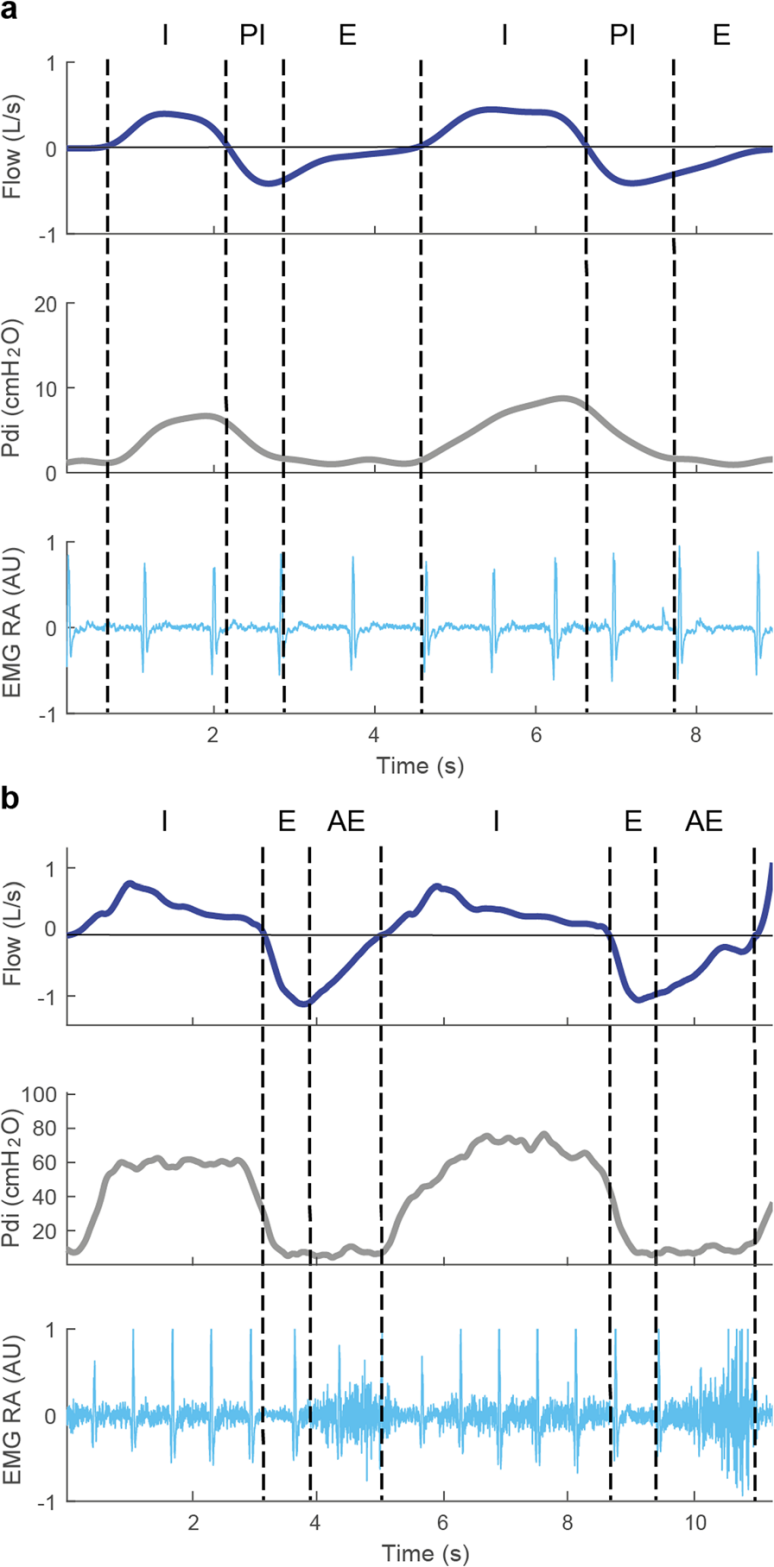
Breathing phases. Flow, transdiaphragmatic pressure (*P*_di_) and electromyography of the rectus abdominal muscle (EMG RA, in arbitrary units; note that this signal is disturbed with electrocardiogram [EKG] artifacts) during tidal breathing at rest (**a**) and during high resistive loading (**b**) in one healthy subject. Vertical dashed lines mark the onset of the different breathing phases. Inspiration (I) is characterized by a steady increase in *P*_di_ and positive flow, and is present during both tidal breathing and high loading. The gradual decrease in *P*_di_ during expiratory flow in (**a**) is consistent with post-inspiration (PI). Note that the rate of decline in *P*_di_ is much more rapid during high loading. During tidal breathing (**a**), expiration (E) is characterized by the absence of *P*_di_ and EMG RA activity and occurs after post-inspiration. High loading (**b**) leads to expiration (AE), which can be recognized by the increase in EMG RA activity. Also, expiration directly follows the inspiratory phase

The respiratory drive originates from clusters of interneurons (respiratory centers) located in the brain stem (Fig. 1) [2]. These centers receive continuous information from sources sensitive to chemical, mechanical, behavioral, and emotional stimuli. The respiratory centers integrate this information and generate a neural signal. The amplitude of this signal determines the mechanical output of the respiratory muscles (and thus tidal volume). The frequency and timing of the neural pattern relates to the breathing frequency and the duration of the different phases of the breathing cycle. Three phases can be distinguished in the human breathing cycle: inspiration, post-inspiration, and expiration (Fig. 2). Each phase is predominately controlled by a specific respiratory center (Fig. 1) [2].

#### Inspiration

Inspiration is an active process that requires neural activation and subsequent contraction (and energy expenditure) of the inspiratory muscles. The pre-Bötzinger complex, a group of interneurons positioned between the ventral respiratory group and the Bötzinger complex in the brain stem (Fig. 1), is the main control center of inspiration [2]. The output from the pre-Bötzinger complex increases gradually during inspiration and rapidly declines when expiration commences. Axons of the pre-Bötzinger complex project to premotor and motor neurons that drive the inspiratory muscles and the muscles of the upper airways. The pre-Bötzinger complex has multiple connections to the other respiratory centers, which is thought to ensure a smooth transition between the different breathing phases and to prevent concomitant activation of opposing muscle groups [6].

#### Post-inspiration

The aptly named post-inspiratory complex controls the transitional phase between inspiration and expiration by reducing expiratory flow. This is achieved by gradually reducing the excitation (and thus contraction) of the inspiratory muscles, which leads to active lengthening (i.e., eccentric contractions) of the diaphragm [2, 7]. Additionally, the post-inspiratory center controls the upper airway muscles. Contraction of the upper airway muscles increases expiratory flow resistance, effectively reducing expiratory flow. Post-inspiratory activity increases the time before the respiratory system reaches end-expiratory lung volume. This can lead to a more laminar expiratory flow and might prevent alveolar collapse, while also increasing the duration of gas exchange in the alveoli [2]. Post-inspiration is a common part of the breathing cycle in healthy subjects at rest, but disappears rapidly when respiratory demands increase, to favor faster expiration [8] (Fig. 2).

The importance of the post-inspiratory phase in mechanically ventilated patients remains unclear, as the onset and duration of inspiratory and expiratory flow depend predominantly on the interplay between ventilator settings (e.g., cycle criteria, breathing frequency, ventilator mode) and the respiratory mechanics of the patient. Additionally, the endotracheal tube bypasses the actions of the upper airway muscles. Experimental data in piglets suggest that post-inspiratory activity of the diaphragm prevents atelectasis and possibly cyclic alveolar recruitment [9], although studies in patients weaning from the ventilator did not find clear evidence for post-inspiratory activity [10]. Clearly, this field requires further research.

#### Expiration

Expiration is generally a passive event during tidal breathing. The elastic recoil pressure of the lungs and chest wall will drive expiratory flow until the lung and chest wall recoil pressures are in equilibrium at functional residual capacity, or at the level of positive end-expiratory pressure (PEEP) in mechanically ventilated patients. In passive conditions, expiratory flow depends solely on the time-constant (i.e., the product of compliance and resistance) of the respiratory system. The expiratory muscles are recruited with high metabolic demands, low inspiratory muscle capacity, increased end-expiratory lung volume, and/or increased expiratory resistance [11].

The lateral parafacial nucleus controls the expiratory phase of breathing. An increased respiratory drive leads to late-expiratory bursts, and consequent recruitment of the expiratory muscles (extensively reviewed in reference [11]). Several inhibitory connections exist between the inspiratory pre-Bötzinger complex and the expiratory lateral parafacial nucleus, which prevent concomitant activation of inspiratory and expiratory muscle groups (Fig. 1) [2, 6].

### Feedback to the Respiratory Control Centers

#### Central Chemoreceptors

The most important chemoreceptors in the central nervous system are positioned on the ventral surface of the medulla and near the ventral parafacial nucleus (also referred to as the retrotrapezoid nucleus). These receptors are sensitive to the hydrogen proton concentration ([H^+^]) of the cerebrospinal fluid (CSF), commonly known as pH [12]. Because CO_2_ can rapidly diffuse across the blood-brain barrier, changes in PaCO_2_ quickly affect the pH of the CSF. A set point exists in the control centers, which keeps pH (and PaCO_2_) within a relatively tight range. A slight increase in PaCO_2_ above this set point provides a powerful stimulus to breathe: a change in PaCO_2_ of 5 mmHg can already double minute ventilation in healthy subjects. When PaCO_2_ decreases only a few mmHg below the set point, the respiratory drive lowers gradually [13] and can abruptly disappear causing apnea, especially during sleep. In contrast, metabolic changes in pH are sensed less rapidly because it takes several hours before the electrolyte composition of the CSF is affected by changes in metabolic acid-base conditions.

#### Peripheral Chemoreceptors

The carotid bodies are positioned close to the carotid bifurcation and are the primary sites sensitive to PO_2_, PCO_2_, and pH of the arterial blood. The aortic bodies contribute to respiratory drive in infants, but their importance in adults is probably minor [14]. The output of the carotid bodies in healthy subjects remains relatively stable over a wide range of PaO_2_ values; their output increases gradually below a PaO_2_ of 80 mmHg and then rises steeply when PaO_2_ falls below 60 mmHg [15]. Their contribution to respiratory drive in healthy subjects is therefore probably modest. However, concomitant hypercapnia and acidosis have a synergistic effect on the response of the carotid bodies, meaning their output is increased by more than the sum of the individual parts. This makes the carotid bodies in theory more relevant in ventilated patients in whom hypoxemia, hypercapnia, and acidosis are more common.

#### Thoracic Receptors

Several receptors have been identified in the chest wall, lungs, respiratory muscles, and airways that provide sensory feedback to the respiratory centers on mechanical and chemical conditions. Slowly adapting stretch receptors and muscle spindles are located in the chest wall, respiratory muscles, upper airways, and terminal bronchioles, and provide information on stretch and volume of the respiratory system, through vagal fibers [2]. These receptors are well known for their contribution to the Hering-Breuer reflexes, which terminate inspiration and facilitate expiration at high tidal volumes (Fig. 1). Irritant receptors line the epithelium of the proximal airways, and are sensitive to irritant gases and local inflammation. These sensors promote mucus production, coughing, and expiration. C-fibers are found inside the lung tissue and might be activated by local congestion causing dyspnea, rapid breathing, and coughing [16].

The relative contribution of these receptors to the respiratory drive of critically ill patients is uncertain. Feedback from these sensors may explain the hyperventilation observed in pulmonary fibrosis, pulmonary edema, interstitial lung disease, and pulmonary embolism, which persists even in the absence of hypoxemia or hypercapnia. Further research into the contribution of these sensors during mechanical ventilation is warranted.

#### Cortical and Emotional Feedback

Stimuli based on emotional and behavioral feedback, originating in the cerebral cortex and hypothalamus, modulate the respiratory drive. Pain, agitation, delirium, and fear are common in mechanically ventilated patients and can increase respiratory drive [17]. The role of the cortex and hypothalamus in the respiratory drive of critically ill patients has rarely been studied and requires more attention before recommendations can be made.

There is some evidence that the cerebral cortex has an inhibitory influence on breathing. Damage to the cortex might dampen this inhibitory effect, which could explain the hyperventilation sometimes observed in patients with severe neurotrauma [18].

## What Is the Effect of Non-physiological Respiratory Drive on My Patients?

### Consequences of Excessive Respiratory Drive

#### Patient Self-Inflicted Lung Injury

Excessive respiratory drive could promote lung injury through several mechanisms. In the absence of (severe) respiratory muscle weakness, high respiratory drive leads to vigorous inspiratory efforts, resulting in injurious lung distending pressures. Recent experimental studies demonstrate that this may worsen lung injury, especially when the underlying injury is more severe [19, 20]. Particularly in patients with acute respiratory failure, large inspiratory efforts could result in global and regional over-distention of alveoli and cyclic recruitment of collapsed lung areas, due to an inhomogeneous and transient transmission of stress and strain (so-called P-SILI) [3, 21]. Large efforts may cause “pendelluft”: air redistributes from nondependent to dependent lung regions, even before the start of mechanical insufflation, and hence without a change in tidal volume [20]. Excessive respiratory drive may overwhelm lung-protective reflexes (e.g., Hering-Breuer inflation-inhibition reflex), which in turn leads to high tidal volumes and promotes further lung injury and inflammation [3]. In addition, large inspiratory efforts could result in negative pressure pulmonary edema, especially in patients with lung injury and/or capillary leaks [21]. As such, a high respiratory drive is potentially harmful in spontaneously breathing mechanically ventilated patients with lung injury. Applying and maintaining a lung-protective ventilation strategy (i.e., low tidal volumes and low plateau pressures) is challenging in these patients and may often lead to the development of patient-ventilator dyssynchronies, such as double-triggering and breath stacking, again leading to high tidal volumes and increased lung stress. Furthermore, maintaining low plateau pressures and low tidal volumes does not guarantee lung-protective ventilation in patients with high respiratory drive.

#### Diaphragm Load-Induced Injury

In non-ventilated patients, excessive inspiratory loading can result in diaphragm fatigue and injury as demonstrated by sarcomere disruption in diaphragm biopsies [5]. Whether this occurs in critically ill ventilated patients is less clear, although we have reported evidence of diaphragm injury, including sarcomere disruption [22]. The concept of load-induced diaphragm injury may explain recent ultrasound findings demonstrating increased diaphragm thickness during the course of mechanical ventilation in patients with high inspiratory efforts [23]. In addition to high breathing effort, patient-ventilator dyssynchronies, especially eccentric (lengthening) contractions, may promote load-induced diaphragm injury [24]. Whether eccentric contractions are sufficiently severe and frequent to contribute to diaphragm injury in intensive care unit (ICU) patients is not yet known.

#### Weaning and Extubation Failure

During ventilator weaning, high ventilatory demands with high respiratory drive increase dyspnea, which is associated with anxiety and impacts weaning outcome [25]. “Air hunger” is probably the most distressing form of dyspnea sensation, which occurs in particular when inspiratory flow rate is insufficient (“flow starvation”), or when tidal volumes are decreased under mechanical ventilation while the PaCO_2_ level is held constant [25]. In patients with decreased respiratory muscle strength and excessive respiratory drive, the muscle’s ability to respond to neural demands is insufficient; dyspnea is then characteristically experienced as a form of excessive breathing effort. Activation of accessory respiratory muscles was found to be strongly related to the intensity of dyspnea [26], and can lead to weaning and/or extubation failure [10]. In addition, dyspnea impacts ICU outcome and may contribute to ICU-related post-traumatic stress disorders.

### Consequences of Low Respiratory Drive

In ventilated patients, a low respiratory drive due to excessive ventilator assistance and/or sedation is a critical contributor to diaphragm weakness. The effects of diaphragm inactivity have been demonstrated both *in vivo* and *in vitro* in the form of myofibrillar atrophy and contractile force reduction [22, 27]. Diaphragm weakness is associated with prolonged ventilator weaning and increased risks of ICU readmission, hospital readmission, and mortality [28]. In addition, low respiratory drive can lead to patient-ventilator dyssynchronies, such as ineffective efforts, central apneas, auto-triggering, and reverse triggering [29]. Excessive ventilator assistance may result in dynamic hyperinflation, particularly in patients with obstructive airway diseases. Dynamic hyperinflation reduces respiratory drive and promotes ineffective efforts (i.e., a patient’s effort becomes insufficient to overcome intrinsic PEEP). Although asynchronies have been associated with worse outcome, whether this is a causal relationship requires further investigation.

## How Can We Assess Respiratory Drive?

Because respiratory center output cannot be measured directly, several indirect measurements have been described to assess respiratory drive. It follows that the more proximal these parameters are to the respiratory centers in the respiratory feedback loop, the better they reflect respiratory drive. This includes, from proximal to distal: diaphragm electromyography, mechanical output of the respiratory muscles, and clinical evaluation.

### Clinical Signs and Breathing Frequency

Clinical signs, such as dyspnea and activation of accessory respiratory muscles, strongly support the presence of high respiratory drive, but do not allow for quantification. Although respiratory drive comprises a frequency component, respiratory rate alone is a rather insensitive parameter for the assessment of respiratory drive; respiratory rate varies within and between subjects, depends on respiratory mechanics, and can be influenced by several factors independent of the status of respiratory drive, such as opioids [30] or the level of pressure support ventilation. We therefore need to evaluate more sensitive parameters of respiratory drive.

### Diaphragm Electrical Activity

Diaphragm electrical activity (EA_di_) reflects the strength of the electrical field produced by the diaphragm and, hence, the relative change in discharge of motor neurons over time. Provided that the neuromuscular transmission and muscle fiber membrane excitability are intact, EA_di_ is a valid measure of phrenic nerve output and thus the most precise estimation of respiratory drive [7, 31]. Real-time recording of the EA_di_ signal is readily available on a specific type of ICU ventilator (Servo–I/U, Maquet, Solna, Sweden). The EA_di_ signal is acquired using a dedicated nasogastric (feeding) catheter with nine ring-shaped electrodes positioned at the level of the diaphragm [31]. Computer algorithms within the ventilator software continuously select the electrode pair that is closest to the diaphragm, and correct for disturbances such as motion artifacts, esophageal peristalsis, and interference from the electrocardiogram or other nearby muscles. EA_di_ reflects crural diaphragm activity and is representative of activity from the costal parts of the diaphragm (and thus the whole diaphragm). In addition, the EA_di_ signal remains reliable at different lung volumes and was found to correlate well with transdiaphragmatic pressure (*P*_di_) in healthy individuals and ICU patients [32, 33]. As respiratory drive comprises both an amplitude and duration component, the inspiratory EA_di_ integral may better reflect respiratory drive than EA_di_ amplitude alone.

#### Reference Values

Normal values for EA_di_ are not yet known, but it is proposed that an amplitude of at least 5 μV per breath in ICU patients is likely sufficient to prevent development of diaphragmatic disuse atrophy [1].

#### Limitations

As EA_di_ amplitude varies considerably between individuals and normal values are unknown, recordings are mainly used to evaluate changes in respiratory drive in the same patient. EA_di_ during tidal breathing is often standardized to respiratory muscle pressure (i.e., neuromechanical efficiency index) [34] or to that observed during a maximum inspiratory contraction (i.e., EA_di%max_) [7]. Although the latter was shown to correlate with the intensity of breathlessness in non-ventilated patients with chronic obstructive pulmonary disease (COPD) [35], it is generally not feasible to perform maximum inspiratory maneuvers in ICU patients. In addition, recruitment of accessory respiratory muscles is not reflected in the EA_di_ signal. Finally, suboptimal filtering of the raw electromyography signal may affect validity to quantify drive with EA_di_ [34].

### Airway Occlusion Pressure

The airway occlusion pressure at 100 ms (*P*_0.1_) is a readily accessible and noninvasive measurement that reflects output of the respiratory centers. The *P*_0.1_ is the static pressure generated by all inspiratory muscles against an occluded airway at 0.1 s after the onset of inspiration. The *P*_0.1_ was described over 40 years ago as an indirect measurement of drive that increases proportionally to an increase in inspiratory CO_2_ and directly depends on neural stimulus (i.e., diaphragm electromyography or phrenic nerve activity) [36]. Advantages of *P*_0.1_ are that short and unexpected occlusions are performed at irregular intervals such that there is no unconscious reaction (normal reaction time is >0.15 s) [36]. Second, the maneuver itself is relatively independent of respiratory mechanics, for the following reasons: (1) *P*_0.1_ starts from end-expiratory lung volume, meaning that the drop in airway pressure is independent of the recoil pressures of the lung or chest wall; (2) since there is no flow during the maneuver, *P*_0.1_ is not affected by flow resistance; and (3) lung volume during an occlusion does not change (with the exception of a small change due to gas decompression), which makes it unlikely that vagal volume-related reflexes or force-velocity relations of the respiratory muscles influence the measured pressure [7, 36]. In addition, the maneuver remains reliable in patients with respiratory muscle weakness [37], and in patients with various levels of intrinsic PEEP and dynamic hyperinflation [38]. Although the latter patient category shows an important delay between the onset of inspiratory activity at the alveolar level (estimated by esophageal pressure [*P*_es_]) and the drop in airway pressure during an end-expiratory occlusion, Conti et al. proved good correlation and clinically acceptable agreement between *P*_0.1_ measured at the mouth and the drop in *P*_es_ at the first 0.1 s of the inspiratory effort (*r* = 0.92, bias 0.3 ± 0.5 cmH_2_O) [38]. The *P*_0.1_ can therefore be considered as a valuable index for the estimation of respiratory drive.

#### Reference Values

During tidal breathing in healthy subjects, *P*_0.1_ varies between 0.5 and 1.5 cmH_2_O with an intrasubject breath-to-breath variability of 50%. Due to this variation, it is recommended to use an average of three or four *P*_0.1_ measures for a reliable estimation of respiratory drive. In stable, non-intubated patients with COPD, *P*_0.1_ values between 2.4 and 5 cmH_2_O have been reported [7], and from 3 to 6 cmH_2_O in patients with acute respiratory distress syndrome (ARDS) receiving mechanical ventilation [39]. An optimal upper threshold for *P*_0.1_ was 3.5 cmH_2_O in mechanically ventilated patients; a *P*_0.1_ above this level is associated with increased respiratory muscle effort (i.e., esophageal pressure-time product [PTP] > 200 cmH_2_O∙s/min [40]).

#### Limitations

Although the *P*_0.1_ is readily available on most modern mechanical ventilators, each ventilator type has a different algorithm to calculate *P*_0.1_; some require manual activation of the maneuver, others continuously display an estimated value based on the ventilator trigger phase (i.e., the measured pressure decrease before the ventilator is triggered, extrapolated to 0.1 s), whether or not averaged over a few consecutive breaths. Considering that the trigger phase is often shorter than 0.05 s, *P*_0.1_ is likely to underestimate true respiratory drive, especially in patients with high drive [39]. The accuracy of the different calculation methods remains to be investigated.

In addition, extra caution is required when interpreting the *P*_0.1_ in patients with expiratory muscle activity; since recruitment of expiratory muscles results in an end-expiratory lung volume that may fall below functional residual capacity, the initial decrease in *P*_0.1_ during the next inspiration may not reflect inspiratory muscle activity solely, but comprises the relaxation of the expiratory muscles and recoil of the chest wall as well [7].

### Inspiratory Effort

Respiratory drive may also be inferred from inspiratory effort measured with esophageal and gastric pressure sensors. The derivative of *P*_di_ (d*P*_di_/d*t*) reflects respiratory drive only if both the neural transmission and diaphragm muscle function are intact. As such, high d*P*_di_/d*t* values reflect high respiratory drive. In healthy subjects, d*P*_di_/d*t* values of 5 cmH_2_O/s are observed during quiet breathing [4]. d*P*_di_/d*t* is often normalized to the maximum *P*_di_, but maximum inspiratory maneuvers are rarely feasible in ventilated ICU patients. A limitation of using *P*_di_-derived parameters is that *P*_di_ is specific to the diaphragm and therefore does not include accessory inspiratory muscles, which are often recruited when respiratory drive is high. Calculating the pressure developed by all inspiratory muscles (*P*_mus_) may overcome this. *P*_mus_ is defined as the difference between *P*_es_ (i.e., surrogate of pleural pressure) and the estimated pressure gradient over the chest wall. Other measurements of inspiratory effort are the work of breathing (WOB), and the PTP, which have been shown to correlate closely with *P*_0.1_ [41, 42]. However, all the above measurements require esophageal manometry, a technique that demands expertise in positioning of the esophageal catheter and interpretation of waveforms, making it less suitable for daily clinical practice. Another major limitation is the risk of underestimating respiratory drive in patients with respiratory muscle weakness; despite a high neural drive, inspiratory effort might be low.

A noninvasive estimate of inspiratory effort can be derived with diaphragm ultrasound. Diaphragm thickening during inspiration (i.e., thickening fraction) has shown fair correlation with the diaphragmatic PTP [43]. However, diaphragm ultrasound does not account for recruitment of accessory inspiratory and expiratory muscles, and the determinants of diaphragm thickening fraction require further investigation. Nonetheless, diaphragm ultrasound is readily available at the bedside, relatively low cost and noninvasive, and may therefore be a potential promising technique for the evaluation of respiratory drive.

## Strategies to Modulate Respiratory Drive

Targeting physiological levels of respiratory drive or breathing effort may limit the impact of inadequate respiratory drive on the lungs, diaphragm, dyspnea sensation, and patient outcome. However, optimal targets and upper safe limits for respiratory drive and inspiratory effort may vary among patients, depending on factors such as the severity and type of lung injury (e.g., inhomogeneity of lung injury), the patient’s maximum diaphragm strength, and the presence and degree of systemic inflammation [3, 19]. In this section, we discuss the role of ventilator support, medication, and extracorporeal CO_2_ removal (ECCO_2_R) as potential clinical strategies for modulation of respiratory drive.

### Modulation of Ventilator Support

Mechanical ventilation provides a unique opportunity to modulate respiratory drive by changing the level of inspiratory assist and PEEP. Ventilator settings directly influence PaO_2_, PaCO_2_, and mechanical deformation of the lungs and thorax, which are the main determinants of respiratory drive. Titrating the level of inspiratory support to obtain adequate respiratory drive and breathing effort might thus be an effective method to prevent the negative consequences of both high and low breathing effort on the lungs and diaphragm [44], although more research is required to determine optimal targets and the impact of such a strategy on patient outcomes.

**Fig. 3:**
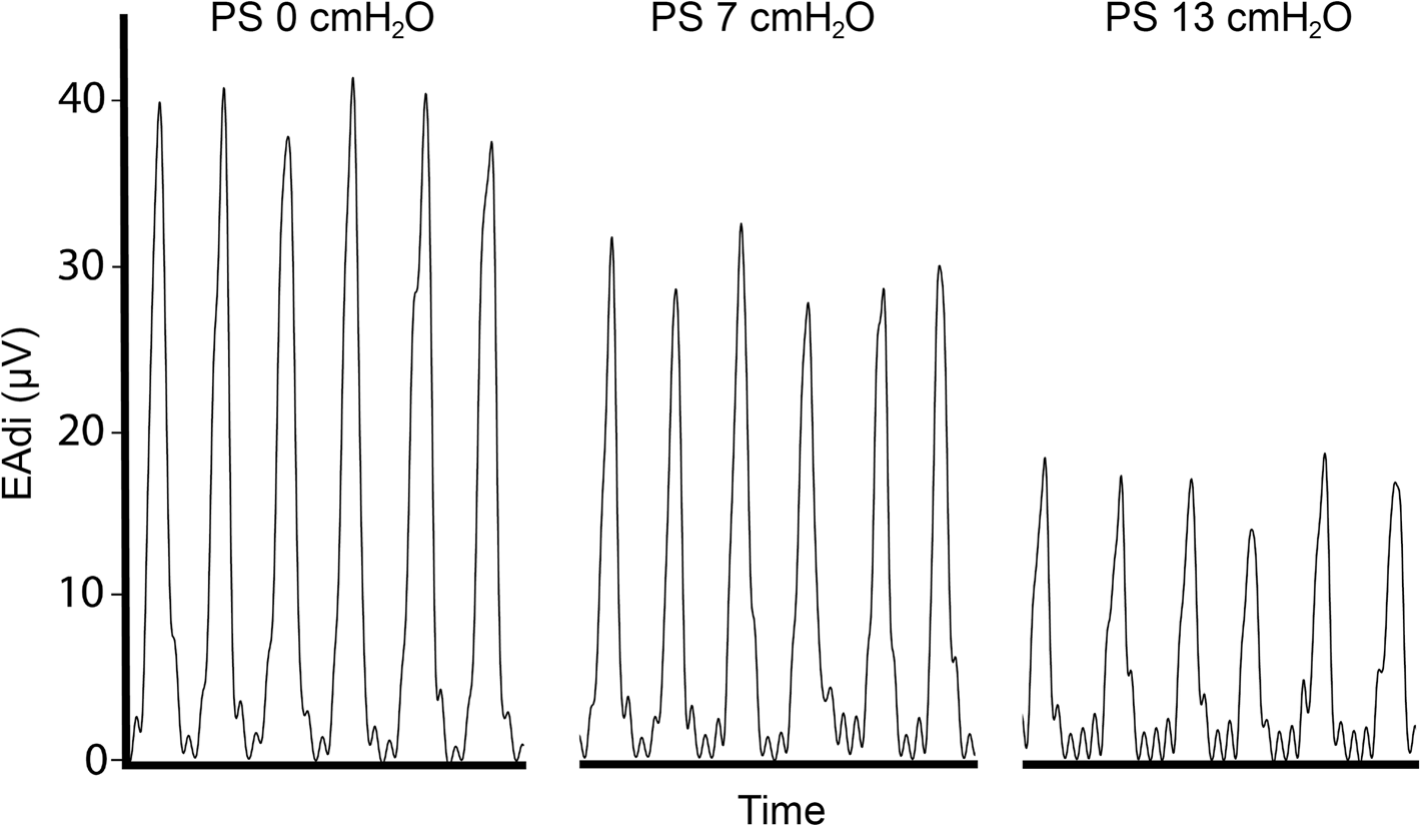
Influence of inspiratory support levels on electrical activity of the diaphragm. Example of a representative patient showing a decrease in electrical activity of the diaphragm (EA_di_, in micro volts) in response to increasing levels of inspiratory pressure support (PS)

Several studies have evaluated the effect of different ventilator support levels on respiratory drive during partially supported mechanical ventilation [45, 46]. Increasing inspiratory support reduces respiratory drive, most evidently seen as reduction in EA_di_ amplitude (Fig. 3) or the force exerted by the respiratory muscles per breath. With high inspiratory assistance the patient’s respiratory effort may even decrease to virtually zero. The respiratory rate seems much less affected by modulation of ventilatory support [4].

If changing inspiratory support level has little to no influence on the patient’s respiratory drive, a clinician should consider whether the elevated respiratory drive originates from irritant receptors in the thorax, agitation, pain, or intracerebral pathologies, and treat accordingly.

### Medication

Drugs can affect the respiratory centers directly, or act by modulating the afferent signals that contribute to respiratory drive [2]. Opioids such as remifentanil act on the μ-receptors in the pre-Bötzinger complex. Remifentanil was shown to reduce the respiratory rate, while having little effect on the amplitude of the respiratory drive [30]. The effect of propofol and benzodiazepines is likely mediated by gammaaminobutyric acid (GABA) receptors, which are widely distributed in the central nervous system. In contrast to opioids, these drugs reduce the amplitude of the respiratory drive while having little effect on respiratory rate [47].

Neuromuscular blocking agents (NMBAs) block the signal transmission at the neuromuscular junction. These agents do not control drive *per se*, but can be used to reduce the mechanical output of the respiratory muscles. High doses of NMBAs completely prevent breathing effort, which might protect against the effects of detrimentally high breathing effort, but could also contribute to diaphragm atrophy [5]. A strategy using low dose NMBA to induce partial neuromuscular blockade allows for effective unloading of the respiratory muscles without causing muscle inactivity. Short-term partial neuromuscular blockade is feasible in ventilated patients [48]. The feasibility and safety of prolonged (24 h) partial neuromuscular blockade and the effects of this strategy on respiratory drive and diaphragm function are currently under investigation (ClinicalTrials.gov Identifier: NCT03646266).

### Extracorporeal CO_2_ Removal

ECCO_2_R (also known as low-flow extracorporeal membrane oxygenation) can be applied to facilitate lung-protective ventilation in patients with hypoxemic failure and respiratory acidosis due to low tidal volumes [49]. ECCO_2_R has been shown to reduce respiratory drive (EA_di_ and *P*_mus_) in patients with ARDS and in patients with acute exacerbation of COPD [49, 50]. The feasibility, safety, and effectiveness of awake ECCO_2_R in patients with acute respiratory failure in order to limit excessive respiratory drive need further investigation. An ECCO_2_R strategy is probably more complex in this group, as the control of drive may be partly independent of PaCO_2_ (e.g., if the Hering-Breuer reflex is overwhelmed), and other organ dysfunctions and sepsis may complicate the clinical picture [49, 50].

## Conclusion

Respiratory drive is the intensity of the output by the respiratory centers and determines the effort of the respiratory muscles. A combination of chemical, mechanical, behavioral, and emotional factors contributes to respiratory drive. High and low respiratory drive in patients under mechanical ventilation may worsen or even cause lung injury and diaphragm injury, and should thus be prevented. Several techniques and interventions are available to monitor and modulate respiratory drive in critically ill patients. The impact of preventing detrimental respiratory drive requires further evaluation, but might be crucial to improve ICU outcomes.

## Data Availability

Not applicable.
